# A national unmet needs assessment for CTSA-affiliated electronic health record data networks: A customer discovery approach

**DOI:** 10.1017/cts.2024.609

**Published:** 2024-10-03

**Authors:** Nallely Mora, Madeline Mehall, Lindsay A. Lennox, Harold A. Pincus, David Charron, Elaine H. Morrato

**Affiliations:** 1 Parkinson School of Health Sciences and Public Health, Loyola University Chicago, Chicago, IL, USA; 2 Institute for Translational Medicine, Loyola University Chicago, Chicago, IL, USA; 3 Colorado Clinical and Translational Sciences Institute, Aurora, CO, USA; 4 Irving Institute for Clinical and Translational Research, Columbia University and New York State Psychiatric Institute, New York, NY, USA; 5 Haas School of Business, NSF and NIH I-Corps Programs, University of California, Berkeley, CA, USA

**Keywords:** Clinical and translational science award, clinical informatics, common data model, data network, real-world data, electronic health records, implementation science, real-world evidence

## Abstract

**Introduction:**

The expansion of electronic health record (EHR) data networks over the last two decades has significantly improved the accessibility and processes around data sharing. However, there lies a gap in meeting the needs of Clinical and Translational Science Award (CTSA) hubs, particularly related to real-world data (RWD) and real-world evidence (RWE).

**Methods:**

We adopted a mixed-methods approach to construct a comprehensive needs assessment that included: (1) A Landscape Context analysis to understand the competitive environment; and (2) Customer Discovery to identify stakeholders and the value proposition related to EHR data networks. Methods included surveys, interviews, and a focus group.

**Results:**

Thirty-two CTSA institutions contributed data for analysis. Fifty-four interviews and one focus group were conducted. The synthesis of our findings pivots around five emergent themes: (1) CTSA segmentation needs vary according to resources; (2) Team science is key for success; (3) Quality of data generates trust in the network; (4) Capacity building is defined differently by researcher career stage and CTSA existing resources; and (5) Researchers’ unmet needs.

**Conclusions:**

Based on the results, EHR data networks like ENACT that would like to meet the expectations of academic research centers within the CTSA consortium need to consider filling the gaps identified by our study: foster team science, improve workforce capacity, achieve data governance trust and efficiency of operation, and aid Learning Health Systems with validating, applying, and scaling the evidence to support quality improvement and high-value care. These findings align with the NIH NCATS Strategic Plan for Data Science.

## Introduction

**Figure 1.**
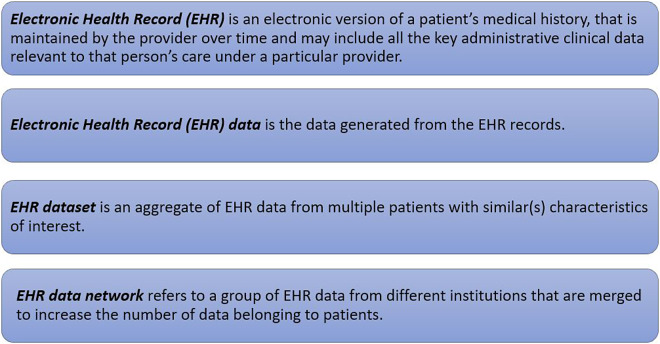
Glossary of terms.

The landscape of Electronic Health Record (EHR) data networks available to academic institutions in the United States (U.S.) has expanded and become more complex over the last two decades. This is partly due to significant national investment resulting from the American Recovery and Reinvestment Act of 2009, including the HITECH Act to promote and expand the adoption of health information technology [1,2] and the establishment of Patient-Centered Outcomes Research Institute (PCORI) and Agency for Healthcare Research (AHRQ)-funded data networks to enable patient-centered comparative effectiveness research [3,4]. Congressional funding further established the Sentinel Network to improve how the U.S. Food and Drug Administration (FDA) evaluates the safety and performance of medical products and currently exceeds 425 million person-years of observational electronic health (EH) data [5,6].Furthermore, the 21st Century Cures Act enacted in 2016 sought to accelerate the discovery, development, and delivery of new therapies aiming to cure prominent diseases using real-world evidence (RWE) from EH data sources for decision-making affecting drug approval and reimbursement [7–9]. A glossary of terms used throughout the manuscript is available in Figure 1.

Most recently, demands for rapid surveillance and outcomes assessment during the COVID-19 pandemic further accelerated proliferation and use of EHR data for clinical and public health decision-making [10].

Since 2011, the National Institutes of Health (NIH), through its National Center for Advancing Translational Sciences (NCATS), as part of other initiatives has funded a national network of academic medical research institutions, now more than 60 hubs, through its Clinical and Translational Science Awards (CTSA) program to advance health informatics research and translation. Specifically, NCATS envisions “progressing toward a standards-based, interoperable network in a cloud environment where informatics assets, e.g., data, software, and algorithms, can be co-developed and shared across the CTSA consortium [11].”

To that end, NCATS has invested in technologies, like the Informatics for Integrating Biology and the Bedside (i2b2) and the Shared Health Research Information Network (SHRINE). i2b2 is an open-source clinical data warehousing and analytics research platform that enables sharing, integration, standardization, and analysis of heterogeneous data from healthcare and research [12]. SHRINE is a web-based query tool that allows investigators to determine the aggregate total number of patients at participating hospitals who meet a given set of inclusion and exclusion criteria. That investment enabled national implementation of the Accrual to Clinical Trials (ACT) Network using SHRINE to enable real-time querying of i2b2-based clinical data warehouses (CDWs) whose data were harmonized with the ACT ontology [13]. The ACT Network was adopted in over 80% of CTSA programs to provide a platform that helps clinical investigators query EHR data, representing over 142 million patients, in real-time cohort discovery to establish the feasibility of a clinical trial protocol for grant applications [14]. State-based and regional data networks have also been enabled by these technologies, such as iTHRIV (Virginia), OneFlorida Data Trust, UCReX (California), and Regenstrief Institute (Indiana) [15–19]. The Observational Medical Outcomes Partnership (OMOP) is another Common Data Model adopted by many CTSAs, including Albert Einstein College of Medicine – Montefiore Health, Columbia University, and Stanford [20,21]. An example of rapid NCATS informatics investment, powered by CTSA collaboration, was the National COVID Cohort Collaborative (N3C) Data Enclave. N3C is one of the largest collections of clinical data from over 60 healthcare institutions across the USA containing data on over 7 million COVID-19 positive cases and related EHR data allowing researchers to study patient outcomes since 2020 [7].

NCATS also strongly encourages CTSA hubs to utilize and leverage existing data resources, such as PCORI-supported National Patient-Centered Clinical Research Network (PCORnet), to promote data reproducibility and generalizability across datasets to further accelerate the translational research process [22]. PCORnet is a fully integrated network that offers RWE on more than 30 million people across the USA through partnerships with Clinical Research Networks. Alternatively in the private sector, there are several EHR database tools also utilized by CTSA hubs, such as TriNetX. TriNetX is a global health research network that provides real-world data (RWD) on over 250 million patients for protocol design and feasibility, site selection, and patient recruitment [23]. Many CTSA hubs contribute data to this network because it provides an easy cohort discovery tool that outperforms other networks’ ease-of-use and efficiency of the clinical trial process utilizing RWD analysis to generate RWE [24,25].

The proliferation of EHR data for clinical and translational research is exciting, and with growing demand, there is increased opportunity to enhance its usability. Moreover, the NIH Strategic Plan for Data Science (2024–2029) emphasizes the optimization of data infrastructure, modernizing harmonization and data ecosystems, accessible data management analytics and tools, enhancing the data-science workforce, and ensuring sustainable data stewardship [26]. Understanding that CTSA hubs vary substantially in their capacity to address informatics challenges in data collection, quality, and harmonization; methodology for deep phenotyping (e.g., discrete and textual data); maintaining patient privacy; ontology harmonization; and ability to transfer data beyond institutional firewalls. Convenient, readily available, and easily accessible tools and solutions that address these problems are required. Moreover, CTSA hubs seek guidance on the complicated governance needed to enable data sharing and analysis of shared data (i.e. Data User Agreement (DUA)); many are seeking to focus limited resources on preferred networks vs. supporting all available. Failure to address these needs stymies collaboration and innovation among CTSA hubs, limits efficient use of funds available for advancing translational science, and ultimately impacts efficient and effective healthcare delivery.

NCATS funded the Evolve to Next-Gen ACT Network (ENACT) to make it easier for investigators at CTSA hubs to conduct impactful research and inform clinical decision-making using real-world EHR data [15,27]. Thus, aligning with the NIH NCATS Strategic Plan for Data Science goals, the ENACT dissemination and evaluation (D&E) workgroup conducted a national needs assessment among CTSAs to better understand the competitive landscape and identify recommendations for ENACT and other RWD EHR networks operating within the CTSA consortium.

## Methods

### Study design

A mixed-methods evaluation was conducted using business start-up principles and methods to assess problem-solution and product-market fit for technology startups [28,29]. Our conceptual framework included designing-for-dissemination-and-sustainability principles from implementation science which incorporates stakeholder feedback early at the stages of product conceptualization and demonstration to enhance Fit-to-Context, for both CTSA hubs and EHR data users at academic research institutions [30]. First, we conducted a Landscape Context analysis to understand the competitive environment, in this case, electronic health data alternatives used for conducting health services research in the USA. [31]. Second, the Customer Discovery method was used to identify strong value proposition(s) for EHR data networks for different customer segments within the CTSA consortium.

For the competitive landscape analysis, EHR was categorized into health survey and surveillance data, EHR data, administrative (claims) data, EHR and administrative (claims) data, multidimensional linked datasets (biorepositories, genomic data, etc.), and trends in RWD like artificial intelligence (AI) and natural language processing (NLP) enabling data. Electronic health data products were subcategorized based on attributes like data governance (public or private), and cost per data acquisition (free vs. fee). (Supplement Appendix 3 and 4)

The customer discovery and value proposition methodology aimed to identify customers/stakeholders’ perceptions of (i) the most important “jobs to be done” for EHR data users; (ii) the critical “pains” and “gains” of different types of EHR data available at their institutions; and (iii) metrics of success as defined by different customers/stakeholders segments [32]. The stakeholders included CTSA hub partners (principal investigators, informaticians, and statisticians), and local end-users (clinical and translational investigators). We employed the I-Corps™@NCATS methodology to inform intervention conceptualization and demonstration for numerous health innovations [14,27,28,33,34]. The process yields a validated message framing about the value of a product idea, a value proposition, from the customer’s perspective in the context of competing alternatives to guide dissemination strategies and promote adoption [30].

### Study team

The study team was comprised of researchers representing four CTSA hubs who are part of the D&E workgroup of the ENACT network. In addition, a national I-Corps instructor, who led the development of the I-Corps™@NIH and I-Corps™@NCATS programs, independently conducted the interviews to reduce confirmation bias. Preliminary results were shared with the project’s Dissemination Advisory Board (DAB) comprised of experts in dissemination research and practice; marketing; and pharmaceutical and health informatics commercialization (See Supplement Appendix 1).

### Study sample

Participants were recruited through a combination of targeted and snowball sampling. Leveraging an existing EHR network (ENACT), we solicited input from principal investigators (PIs) for each CTSA hub enlisted in the network via survey. Participation was voluntary and requested via email by the ENACT leadership team. Survey data included names of identified stakeholders for RWD research databases at each institution for soliciting interviews and relevant referrals. Additionally, we conducted a literature review to identify publications that used EHR networks like ACT, N3C, and TriNetX. Authors belonging to a CTSA hub were contacted soliciting an interview.

#### Ethical considerations

The study was determined exempt by Loyola University Chicago’s Institutional Review Board (LU IRB #217691110723). Interviewees gave verbal consent before the interview. Participants were not compensated.

### Data collection

Data were collected via survey, interview, and focus group, between February and May 2023.

#### Survey

The survey included public data elements such as institution name, names of the CTSA hub PI, and key personnel including leaders from informatics; learning healthcare systems; statistics; and other researchers that utilized RWD at their institution. We did not collect additional personal information.

#### Interviews

The main objective was to gather participants’ input on the existing EHR data sources at their institutions. The interview guide followed the customer discovery framework to learn about current practices, perceived barriers, and facilitators concerning RWD utilization for research and translational science based on their roles in the academic healthcare ecosystem. We targeted to conduct 50 interviews, based on the I-Corps™@NCATS program recommendations.

Questions were open-ended and adjusted in an iterative process (Interview Guide Supplement Appendix 2). One-on-one interviews were conducted via phone or virtually (Zoom), with an average duration of 40 minutes. Zoom interviews were recorded and transcribed by Otter AI software (https://otter.ai/). Customer discovery team debriefed after interviews.

#### Focus group

A 1.5-hour focus group was conducted with informaticists and health informatics researchers interested in RWD/RWE research in the CTSA environment. Structured questions about health informatics’ future, barriers, and facilitators for achieving informatics RWD goals guided the discussion. (Supplement Appendix 2).

### Data analysis

Survey data-informed descriptive statistics, including participating CTSA hubs and roles of interviewees per site. Analysis was supported by Excel software.

#### Interview and focus group data

Transcripts and notes from interviews served as the primary data source for analysis. Interview transcripts were analyzed by three investigators (DC, NM, MM) using rapid qualitative analysis methods, which involved: (1) creating a matrix for codes, (2) establishing interrater reliability by independently coding three interviews and generating consensus, (3) independently coding the remaining interviews, and (4) summarizing themes [35]. Following the customer discovery and value proposition framework relevant themes were identified and grouped by interviewee role [29,32]. Qualitative analysis was supported by QSR International Pty Ltd (2020) NVivo 12 software (lumivero.com). The research team, customer discovery team, and DAB met regularly to discuss findings.

## Results

### Landscape Analysis

**Figure 2.**
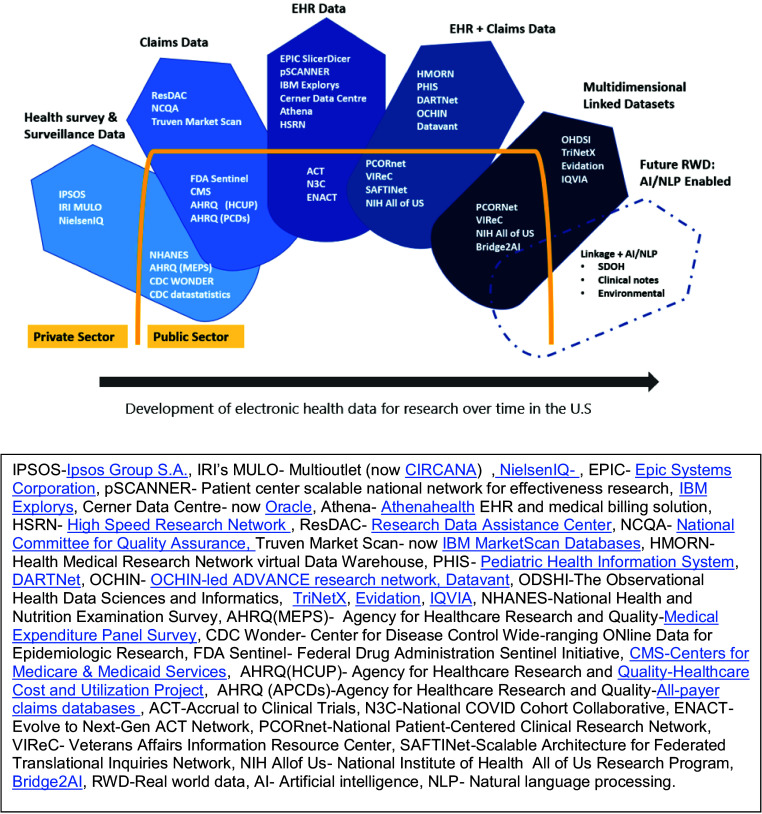
Landscape of alternative electronic health datasets available in the USA for research.

Figure 2 shows the landscape of an alternative range of public and private EHR data by types of data (administrative claims, EHR, linked data) that have evolved and are available in the USA. The list of available options is large and requires expertise to know which datasets are convenient and abie to answer specific research questions. The attributes considered during the competitive landscape analysis were governance (open access vs. membership), cost, and scale (national vs. international), information was obtained from the websites and existing literature (Supplement Appendix 3). We identified comparable CTSA-based EHR data networks, that are RWD public sources, like ACT, N3C, and ENACT. Other relevant product attributes are specified in Supplement Appendix 4, representing the competitive alternatives for researchers using EHR RWD within the CTSA ecosystem. The product gap analysis showed that EH data products available to CTSA hubs are numerous.

### Customer discovery findings

A total of 32 institutions with membership to 27 CTSA hubs participated in the survey and/or interview process, approximating a 57% participation response rate of the current participating ENACT network (57 total). Between April and August 2023, we conducted 54 one-on-one interviews and one focus group with seven individuals, adding up to 61 individuals contributing to qualitative data for analysis. Table 1 shows the study population characteristics by CTSA hub contributing to data.

**Table 1.** Characteristics of the study population by CTSA sites contributing to interview and/or focus group data

Site		Role			
	N	Researcher	Informaticist	Statistician	CTSA PI*
**Total**	**61**	**20**	**16**	**17**	**8**
Columbia University, New York	1	1			
Harvard Catalyst, Harvard University	4		2	2	
Indiana University	1			1	
Medical University South Carolina	1	1			
Oregon Health & Science University (OHSU)	1		1		
Pennsylvania State University	5	1	3		1
University of Michigan	2	2			
University of Arkansas Medical Sciences	1	1			
University of California Los Angeles (UCLA)	2	1		1	
University of California San Diego (UCSD)	1			1	
University of California San Francisco (UCSF)	3		2	1	
University of Chicago (Institute Translational Medicine)	6	3		1	2
University of Cincinnati	4	1		2	1
University of Colorado Denver	1			1	
University of Florida	1	1			
University of Minnesota	2	1		1	
University of North Carolina	1			1	
University of Pittsburgh	1	1			
University of Texas Medical Branch	2		1		1
University of Utah	3	1	1		1
University of Virginia	4	1	1	1	1
University of Washington	3	2			1
University of Wisconsin	1	1			
University of Wisconsin-Madison	2		2		
Wake Forest University	4	1		3	
Washington University St Louis	2		2		
Weill Cornell Medicine, New York	2		1	1	

*CTSA PI = CTSA principal investigator.

The 61 interviewees were comprised of 8 CTSA PIs, 16 Informaticists, 17 Statisticians, and 20 Researchers. Some individuals “crossed over” between groups, representing different points of view. For allocation purposes, the role represented in the descriptive analysis is how it was identified by their CTSA hub.

The next section describes the findings from the data analysis obtained through customer discovery methodology, results are organized by five emerging themes, that informed the value proposition for each customer segment. Illustrative quotes and value propositions by customer segmentation are described in detail in Tables 2 and 3.

**Table 2.** Summary of customer discovery findings describing value proposition by CTSA segment

Customer discovery findings	CTSA hub*	
	Small*	Large/medium
**Jobs to be done**	Have a reliable, accessible, and friendly user RWD network	Endorse a preferred network
**Pains to be solved**	Need funding to support and maintain the RWD Network platform	Some are seeking much greater central support
**Gains to be achieved**	To have an established network that facilitates interdisciplinary collaboration	This reduces the burden on the CTSA Hubs themselves.
**Illustrative quotes**	“We have a lot of needs. And we have two large networks that are currently unfunded, this being one of them. And so, we’re trying to determine where we have ability to provide staff from our infrastructure, baseline funding from the hospital, versus what we might have to forego because of that.”	“We do need a core service amongst our investigators.”
	“On the federated side, you do have to have more infrastructure.”	“This week, I had 40 inquiries for support, and I touched 100 different networks.”
	“… [without funding support] not necessarily a ton of incentive for sites to improve or maintain the quality of their data.”	“… until we have a bigger bench of individuals who have the appropriate training and clinical bioinformatics, we need this is as a core service.”
**Targeted value proposition**	For small CTSAs that need an accessible, user-friendly Real World Data network and connections with skilled data science collaborators. The EHR Network…	For CTSAs that need better operational efficiency for curating and analyzing Real World Data the EHR Network… …
		… provide a Centralized Support Center that will enable them to have access to data quality support and training resources better than or like national commercially supported data vendors …
	… provide data infrastructure support and a Centralized Support Center that supports data sharing capacity while enabling access to collaborators and people with skills better than or like their local EHR data source …	
	… provide the benefit of better collaborative RWD team formation that can utilize an established national CTSA-friendly network for generalizable clinical and translational research	… provide the benefit of reducing the local burden of sustaining multiple collaborative RWD networks across CTSA hubs

RWD = Real-World Data; CTSA- =Clinical Translational Science Award; RWE = Real World Evidence.

*CTSA hub size based on NIH NCATS award < $30M designated as small, and ≥ $30M large/medium.

**Table 3.** CTSA customer segmentation in relation to real-world data use for clinical and translational research

Customer Type	Small CTSA	Medium CTSA	Large CTSA
**User: Researchers** The clinical or public health investigator with subject matter expertise	Most researchers are not skilled. Those that are, are finding a way on their own.	A mix of researchers begins to appear with some having combined skills	A wide array of new and old, skilled, and unskilled faculty exists.
**User: Informatics** The researcher with data science expertise	Low budget, small staff, operations with the need to train their researchers.	Core facilities start to emerge and develop their own business models and preferences.	These can be high volume and start to take control, via influence, of the research tools.
**User: Statistics** The researcher with biostatistics, epidemiology, and/or econometric expertise	Across all institutions, when available, these individuals have a strong influence over research design and cohort development. Some researchers take on the statistical analysis on their own and stick to existing databases.		
**Buyer: EHR Data Acquisition** The person who identifies which data networks to purchase and/or support	Grant and budget-focused	CTSA members who are stretched for budget.	CTSA facilities that have figured out their business model
**Influencers** The people who influence the local business model and data governance necessary to advance local health informatics research	None	Attorneys and administrators of all stripes	Researchers with grant funding that core facilities tax
**Decision Makers** The person who ultimately decides which electronic health networks to support and sustain for local clinical and translational research	Appointed leads	Many	Research tool managers, leaders, and others who have the benefit of grant dollars

*CTSA hub size based on award <$30M designated as small, $30M to $49M as medium, and ≥ $50M large.

#### CTSA segmentation needs vary according to resources

We created an ecosystem mapping to identify our target customer within the CTSA health informatics ecosystem (organizational structures, influencers, and incentives), to identify the “end users” and characterize CTSA archetypes that will help to target specific customer segments to develop EHR network sustainability strategies. (Table 3 and Supplement Appendix 5).

The findings on customer segmentation related to RWD within the CTSA environment identified two archetypes of CTSA hubs: large-medium and small CTSA hub size based on award < $30M designated as small, ≥ $30M large/medium, reflecting the number of resources they might have to devote to EHR data acquisition, sustainment, and training. Stakeholders were categorized by: (a) End users: researchers, informaticians, and statisticians; (b) Influencers: CTSA hub PIs and cluster leaders; and (c) Decision makers: CTSA hub PIs and leads of clinical research offices.

Results show that the interest and capacity to support large, federated data networks varied between CTSAs, specifically according to the institution size, system support, and funding. Highly resourced (large) CTSAs have greater capacity to “add one more network” and often intentionally belong to many vs. endorsing a preferred network. In contrast, low-resourced (small) CTSAs need funding to adopt and maintain the EHR network platform.

Small institutions are focused on allocation of budget and resources according to grant cycle and success, and researchers need more resources to perform their research with RWD. Therefore, small institutions learn how to operate with small budgets and low staff and resources.

*“I know enough to be dangerous, but I do not know enough to analyze those large datasets.” -Clinical Researcher.*

In medium-sized institutions, there is a mixture of researchers that begin to appear with some combined skills and roles in the institution (clinician and informatics). Some have an informatics team that helps support researchers; however, budgets are still constrained and grant-dependent.

*“We’re still debating about no funding for “X” network. We have a lot of needs. And we have two large networks currently unfunded, this being one of them. And so, we’re trying to determine where we have the ability to provide staff from our infrastructure, baseline funding from the hospital, versus what we might have to forego because of that.” -CTSA PI.*

Large institutions tend to have well-defined teams and workgroups that support others in the research process. The range of RWD researchers is wide, ranging from senior to new and skilled to novice. Support resources are available, but there are still concerns about capacity building. Local influencers-decision makers are those who have successfully secured grant dollars.

*“Until we have a bigger bench of individuals who have the appropriate training and clinical bioinformatics, we need this as a core service.” -CTSA PI, researcher.*

Workflow and decision-making on research activities involving RWD vary between CTSA hubs. The medium to large institutions’ process starts with the main researcher with research idea generation and search for feasibility of the study by the PI, which is when they start considering the cohort size for the project. The main influencers are the existing collaborator network mostly defined by the research line, subsequently, the collaborators suggest an RWD network they are familiar with and reach out to the core facility to explore or ask for suggestions, being those core leaders or staff advising the decision makers on what dataset will be used. Thereafter, the statistician weighs in and influences if the dataset will be adequate to answer research questions, or if necessary to re-scope data source. Across all institutions, when available, these individuals have strong influence over research design and cohort development. Some researchers take on the statistical analysis on their own and stick to existing databases. Supplemental Appendix 5 shows the ecosystem map of workflow.

*“At least for my group is a team-based effort, because I may simply not even know that database exists. But somebody on the team might know and have access and expertise.” -Researcher, Informaticist.*

#### Team science is key to success

Many interviewees highlighted the importance of teaming to be successful with research involving RWD, which at a minimum includes a researcher, informatician, and statistician. The team science approach was a consistent concept among all CTSA sizes and remarks the importance of having a multidisciplinary collaboration.

*“A multidisciplinary team…It’s these large groups that are the most successful at accessing [and analyzing] the data.” -CTSA PI.*

#### Quality of data generates trust in the network

Data quality and types of data requirements and expectations vary significantly between types of clinical and translational researchers.

*“We [can] broadly group people into investigators who know they need data and know how to use it … the investigators who appreciate that they could probably benefit from data, but they do not know how to use it. … And there are those who do not even realize that they need data and would benefit from using it. And most often, they also do not know how to use it anyway.” -CTSA PI.*

Methodologically skilled researchers (e.g., clinical/pharmaco epidemiologists, health services researchers, and informaticists) conduct sophisticated hypothesis-testing research which requires greater data granularity than an aggregated de-identified dataset provides. They are comfortable seeking and using other available datasets, so they place higher value on quality of the data than on ease of access to the data.

*“The whole issue comes down to the quality of the data available, and the ability to access the data and by quality.” -CTSA PI, Informaticist.*

#### Capacity building is defined differently by researcher’s career stage and CTSA’s existing resources

The need to build capacity and foster collaboration was a consistent theme in our interviews with potential users of federated databases. Most researchers utilize data from their own local and trusted sources or collaborators. The need lies in capacity-building catalysts training tools, and a place to scale when a big sample size is needed (“Big N”). The interviews often were satisficing in their dataset decisions. Just enough data to publish was sufficient, and the benefit of extending that dataset to a “Big N” was not compelling even when they might have been able to access it through a national network.

*“It’s the institutions and collaborations, and then you try to if you can make the data work.” -Informaticist.*

Novice data science researchers either want to learn the skills (e.g., graduate students/fellows) or outsource the skills concierge-style (e.g., clinical investigators).

*“[Training] It’s a substantial time investment that I don’t currently have.” -CTSA PI, researcher.*

#### Researchers’ unmet needs

There are currently unserved researchers – e.g., public health-population health, implementation science, and learning health system investigators – who could represent a “new target segment,” an opportunity for new use cases and scale-up of existing EHR networks. A network to investigate the implementation of policies and quality indicators would also have interest, networks that allow a collaborative learning health system. This taps into different communities within the CTSAs to expand their user base and reach.

*“Build systems that enable new capabilities, solve problems/issues and are interoperable and reusable in other places.” – Researcher, Informaticist*

A desired feature was the ability to perform descriptive statistics and explore variables on the same platform.

*“We want to check a few features to see whether they are by a known bias for example, population times demographic features, whether they are bias and the longitudinal data availability.” -Researcher*

A clinical decision support network emerged as a strong unmet data need – specifically, a sandbox to validate generalizability of something developed locally and test its scaling. Many seek a “GitHub”-like agnostic learning network to share informatics tools and aggregate data “add-ons,” making the process easier.

*“Having access to source code is the best thing.” -Informaticist*

Future desired features included the addition of clinical notes by using AI and NLP, which calls to adapt existing data networks, some institutions are already working on those applications and integration to existing EHR.

*“But then there’s more data, meaning like …maybe you can extract content concepts from clinical notes, or link to images or other kinds of things…making the network more useful.” -Researcher.*

With the insights from our customer discovery, we developed a value proposition for each customer segment. Table 4 summarizes the Value Proposition by attributes and customer (stakeholder) segmentation. One of the benefits of generating a value proposition is to define the “Minimum Viable Product” (MVP), which is the smallest amount of product features and capabilities necessary for release, and then to slowly add more functionality as needed to a new product while collecting validated learnings in the process [33,36]. The MVP concerning EHR database networks was the same across customer segments (stakeholders).

**Table 4.** Value proposition by attributes and customer segment

Value Proposition		Customer Segment			
		PI/Informatics Cluster leader		Researcher	Informaticist
**The RWD federated data network for academic researchers…**		… provides a CTSA-wide collaborative network with core support center services, with quality data to conduct observational studies.		… provides easy-access, credible data for observational studies	… provides a credible, harmonized RWD for research
**Attribute: Valid and credible data source**	**What I need…**	Trust in the data-governance, process, and quality of data…		Transparency on publications	Quality of the data (curation, maintenance)
	**Illustrative quotes**	*“And if we’re not actively monitoring that data, then we do not know if it’s still valid. And so that’s usually the biggest reason why I do not have people use networks is because I do not feel confident in there. And it’s data quality.”*		*“Reporting of the model has to be transparent about what methods you use, you know, the type of data that you have, and it cannot just be like a general description, it should be something that gives people a sense of like, okay, this is, you know, the cohort that I selected, what were the inclusion-exclusion criteria, you know, things like that, that’s still not as good as just getting to the actual, you know, code.”*	*“If you do not have enough users who are on it, or who understand the incentive, then institutions do not have any incentive to sort of like, keep a high-quality node active, then you get, you know, a more inconsistent set of responses, less accurate data, then it’s less useful for users, etc.”*
			*“The whole issue comes down to the quality of the data available, and the ability to access the data and by quality, I mean, how well it’s curated, how robust it is in terms of numbers, and how much information and provides about.”*	*“Having access to source code is the best thing. And if we can get there, that would be great. But if not, then I think better model reporting is important.”*	*“Having a team that is doing data management, so we know the data is high quality.”*
	**What evidence will convince me…**	An active monitoring data system in place (model PCORnet)		Have access to open-source datasets, repositories, and data models and be able to share results	When there is an increase in enthusiasm and return of users
**Attribute: Training support center**	**What I need…**	A central team for quality control and facilitate collaboration		A coordinating center with services	A collaboration space
	**Illustrative quotes**	*“There’s a linking problem there, we have to be able to connect the informatics people with the clinicians that have the research questions, and with the biostatisticians, who can help out with the analysis.”*		*“I know enough to be dangerous, but I don’t know enough to like analyze those large datasets.”*	*“Definitely, that definitely some collaboration space or uploading, make it a natural process.”*
		*“it’s one thing to have someone who is capable of the technical part. But then you need someone who is fluent enough in medical terminology.”*		*“Where I think that there’s a lot of space to improve, and where I could see benefit and moving is really towards the ability to do secondary data analysis. A lot of times that secondary use, it’s going to happen before that clinical trial… that that would be a big benefit.”*	*“So, to some extent, having letting having no central kind of data management, allows sites to have control of their data, active set up to have some flexibility within the network. So, sites could have different data locally, and it can still all work together.”*
	**What evidence will convince me…**	When there is access to an interdisciplinary collaboration center		Short term: number of projects supported, grants, papers. Long-term: health outcomes (impact)	When there is continuous improvement
**Attribute: Efficient and friendly use**	**What I need…**	Affordable service and funding with less regulatory barriers		Data linkage to other data sources (i.e., images, clinical notes, SDOH)	Extraction software for clinical notes, and real data analysis
	**Illustrative quotes**	*“[regarding existing databases]. They got to see the value and be willing to volunteer the effort to installing and implementing and supporting this.”*		*“But then there’s more data, meaning like, there’s basic EHR data, but maybe you can extract content concepts from clinical notes, or link to images or other kinds of things. That’s more data and making the network more useful.”*	*“Having an easy way for me to just put this model somewhere, and then have it run across different sites would be helpful.”*
		*“I do my go to database, because again, I have people who have expertise in it. And because we have free access, that is already IRB approved…”*		*“So, I really care where it comes from for how easy is it going to be to work with?”*	*“And so that’s really where I think it becomes useful is being able to use those advanced analytical tools and create your own analysis.”*
	**What evidence will convince me…**	when there is buy-in from institutional leaders and investment		Able to transfer data from institutions, in friendly and easy access	To be able to transform data from model to model (i2b2 to OMOP)

*“A supportive governance structure, data quality, efficient and easy to use, and supported by a training center that will foster skills acquisition and collaboration across the network”*

However, there was a difference in how these benefits needed to be demonstrated by customer type. The respondents indicated that use cases were essential to prove the value of the RWD network available versus current practice and competing alternatives.

## Discussion

A wealth of EHR data networks is available to clinical and translational researchers at US academic institutions. Over the past two decades, the number and accessibility of these networks have increased, infrastructure and processes for access and data governance have improved, and both informatics tools and informatics departments have proliferated at US academic medical centers. Trends indicate that data integration with imaging, laboratory results, and harmonization among different institutions are in order. Some networks are currently working on integrating social determinants of health (SDOH) data into existing platforms to enable large-scale data analysis for research (i.e., Sociome Data Commons) [37].

Given the plethora of options, we believe our systematic evaluation of unmet needs amongst CTSA hubs and clinical and translational scientists is timely. Moreover, the development of new EHR data networks must include a sustainability plan grounded in clearly articulating a differentiating value proposition relative to existing options. The I-Corps@NCATS customer discovery approach is an implementation science methodology well-suited for quickly uncovering unmet needs, elucidating a network’s value proposition, and understanding evolving “fit-to-context” requirements necessary for adoption and sustainability [30].

Some networks have published their lessons learned to inform dissemination and sustainability strategies [24,27,34,38]. Their results show three prominent themes: the importance of data network maturity (governance, quality, and management), commercial viability considerations as proxy for sustainability, and stakeholder support among data stewards, informaticists, and researchers. However, the emergence of new networks with new attributes changes the market, and as end users’ needs evolve, sustainability strategies must be reevaluated continuously.

With that spirit, our group conducted a needs assessment on EHR data networks among academic research centers within the CTSA environment to understand the current competitive landscape and unmet needs to identify recommendations for RWD networks.

Our key findings are that there is no need for a new dataset, major players government-non-profit (Sentinel, PCORNet, VA, OCHIN) and private sector (EPIC, Oracle, IBM, TriNetX, IQVIA) are leading, and competing, in this area. Therefore, the clinical and translational science gap is in the following areas: (1) Team science – and new methodological approaches to test, validate generalizability, and to implement and scale to impact healthcare delivery and public health decision-making; (2) Data governance trust and efficiency of operation (in the context of ever-evolving cyber security) – we are now at the stage of ’economies of scale’; (3) Enable learning health systems (LHS) that can generate, adopt, and apply the evidence to support quality improvement (QI) and high-value care (Computer systems/AI, continuous QI); and RWD/RWE (FDA, pricing/reimbursement), details in Table 5.

**Table 5.** Expectations for EHR data networks within CTSA academic health centers

Need(s)	Implication(s)	Illustrative Quote(s)
Team Science	The current method is to connect with a friend-colleague and then find the data. Not search the data and then find a friend-colleague. A matching service would be of value. For example, someone’s local CTSA may not have colleagues interested in an investigator’s particular area of interest, or already is heavily saturated so it’s difficult for a junior investigator to build an independent line of investigation.	*“At least for my group is a team-based effort, because I may simply not even know that database exists. But somebody on the team might know and have access and expertise. So, it really, we try to bring people from different walks of life, and different expertise as collaborators.” –Informaticist*
		*“We do not want to sort of reinvent the wheel over and over again. And so when and where possible, we try and sort of converge across teams or across different bodies of work on that kind of definition.” – Informatics core manager, researcher*
Workforce Capacity Development	Centralized training and a learning lab. Not just for CTSAs themselves, but a learning community that can plug this curriculum into graduate education to meet competencies. This could be accomplished by maintaining the EHR data network with just a small subset of institutions.	*“… until we have a bigger bench of individuals who are have the appropriate training and clinical bioinformatics, we need this is as a core service.” – CTSA PI*
Governance	For CTSAs that do not have a robust set of EHR data networks already, there is a segment desiring easy access for exploration.	*“I do my go-to database, because again, I have people who have expertise in it. And because we have free access, that is already IRB approved.” – Researcher*
Data Linkage	RWD has many sources, the integration for a more in-depth analysis of health conditions is necessary. Many CTSAs are linking social determinants of health with their local data in response to calls for health equity research. NIH is also beginning to put out RFAs for data-linkage R01s.	*“The more these networks can connect, and are built on a harmonized platform, the larger populations, and the more utility they have.” – Data Scientist*
Clinical Decision Support	Scale and test the generalizability of locally developed decision tools. CTSAs are actively developing algorithms and tools locally. They know they need to test-validate in more diverse and varied populations. This aligns very well with NCATS’ interests.	*“But then there’s more data, meaning like, there’s basic EHR data, but maybe you can extract content concepts from clinical notes, or link to images or other kinds of things. That’s more data and making the network more useful.” – Researcher*
		*“So, I cannot get medication doses, for example, which makes it hard to think about real analysis for risk safety, side effects, etc.” -Clinical Researcher.*
Learning Health Systems	There is a group of researchers with unmet needs that represent an opportunity for EHR data networks. For them, easy access to national data is appealing. They would be less concerned about the number of patients and more interested in the number of CTSAs and having them represent a diverse set across the country – like the Cancer SEER sentinel network. A network to investigate the implementation of policies and quality indicators would also have interest, networks that allow a collaborative learning health system. This taps into different communities within the CTSAs to expand their user base and reach.	*“Build systems that enable new capabilities, solve problems/issues and are interoperable and reusable in other places.” – Researcher, Informaticist*
		*“Enable operation systems, with people around quality improvement to increase revenue and meaningful to health care system.” – Informaticist*
		*“There’s something really appealing about you know like EPIC has to offer where, where they can take that sort of aggregate query and actually execute it on our system locally and allow us to do things, not just in the abstract, but in the in the production health record with that data, that helps drive care forward in in ways that I think are really important for us.” – Informatics Core Leader*

Health informatics translational research involving RWD is a team sport requiring expertise in data informatics, statistical analysis, and the subject matter (clinical or public health). Forming and sustaining research teams, especially when one of the areas of expertise is limited at your local site, is a key translational hurdle and pain. According to our findings, a team science approach positively impacts translational research, a multidisciplinary collaboration that at minimum requires a researcher, an informaticist, and a statistician. Collaboration with other institutions is desirable but limited. Therefore a “matching service” and/or improving workforce capacity and development by centralized training and learning labs will support the need and fill the gap.

Our interview findings were that centralized networks were favored over federated networks for reasons like trust in quality, local regulatory (IRB, DUA) awareness-experience, and familiarity within the research teams. Reflections of our learnings include that while there is an interest in joining a federated network among academic research centers, governance is a barrier. Most institutions want to retain their data within institutional boundaries, under the perception of saving time and effort required to obtain necessary legal and other approvals (IRB, DUA). Elements identified as important when belonging to federated networks are (i) The ease of inter-member data sharing; (ii) No cost and data visualization software and hardware for institutional use; and (iii) Support and opportunity for collaboration.

Trust plays a significant role when selecting a data network. Access to large quantities of clinical data from operational EHRs holds much promise, however, a major concern is that data not collected systematically for research will be of poorer quality, introducing bias to findings generated from these data. Elements of trust included data provenance, quality control processes, data feed, and availability to check for clinical parameters. Data quality has been addressed previously by other data networks by assessing the conformance, completeness, and plausibility of each contributing organization’s data against the network [39–41]. RWD networks like PCORNet and TriNetX integrate data quality as part of their services, gaining trust from users, by using centralized checkpoints to surface site-to-site differences and optimize data quality [42].

Data science and health informatics are some of the commonly used methods by LHS with the overarching goal of enabling LHS scientists to design, conduct, implement, disseminate, and scale patient-centered outcomes research to improve the quality of care and patient outcomes in diverse health systems. With that healthcare organizations are applying the evidence to support QI and high-value care. However, not all organizations have the same resources, which is why belonging to a network that amplifies the data sources is important. Our findings indicate that people are excited about the concepts of “learning health systems” or “evidence-generating health systems.” However, these concepts were left largely undefined, but the directionality was clear - bridging the clinic and the existing databases into a system. There was recognition that the field is evolving because technical capabilities are still changing. Expressed as: (a) This change unlocks many research opportunities, (b) The existing trouble hiring the people to execute those opportunities due to commercial companies paying more than academic healthcare centers.

Overall, our findings are consistent with operational factors described when building and operating a data network to attain success including (i) purpose, governance, and funding; (ii) network management oversight; (iii) analytic approach and querying technology; (iv) privacy, confidentiality, and security; and (v) transparency [25]. Addressing these factors will impact the success and sustainability of the data network. Sustaining high-value RWD networks is costly and resource-consuming, and federal funding is unsuited to sustaining these initiatives beyond the awarded grant cycle.

Academic institutions frequently face scarce resources to sustain data networks, and often have competing interests and priorities, as well as other available opportunities for “free.” The NIH Strategic Plan for Data Science indicates that the growing cost of managing data is a pressing issue in the data-resource ecosystem, especially in “siloed” centers that do not support data sharing. Specifically planning carefully how resources are to be spent efficiently is necessary to obtain most benefit from investments [26]. An option is to partner with the private sector to advance translational technologies, bringing private-sector expertise, financial and technical resources to achieve the ultimate goal of improving patients’ health. There is opportunity to marry informatics and data science research expertise available in academic networks, like CTSA consortiums, with the dissemination and user-experience resources available with private sector EHR data networks (i.e., EPIC, TriNetX), allowing translational science to move forward effectively. However, inherent conflicts of interest may make that challenging.

The customer discovery methodology allowed us to understand points of view of different customer segments enabling the development of an MVP for EHR data networks. Addressing the minimal needs of end users of EHR data networks is just the beginning of a sustainable product. As described previously, the sustainment of a network relies on transparent network governance, trustworthy data, shareability, collaboration of the network and training, and infrastructure maintenance funding [24,28,34]. In addition, it is important to collectively envision short-term and long-term success metrics, and build use cases to fortify the network, secure ROI, and demonstrate worthiness for new users to build the community.

## Conclusion

Academic research centers belonging to the national CTSA consortium are crucial to achieving the goals of the NIH NCATS Strategic Plan for Data Science, specifically, developing innovative partnerships and collaborations with a strategic array of stakeholders to achieve long-term sustainability. EHR data networks like ENACT that would like to meet the expectations of academic research centers within the CTSA consortium should consider filling the gaps identified by our study: foster team science, improve workforce capacity, achieve data governance trust and efficiency of operation, and enable LHS that can generate, adopt, and apply the evidence to support QI and high-value care.

## Supporting information

Mora et al. supplementary material Mora et al. supplementary material

## Supplementary material

The supplementary material for this article can be found at https://doi.org/10.1017/cts.2024.609.
