# Supplementary material for: A national unmet needs assessment for CTSA-affiliated electronic health record data networks: A customer discovery approach
Source: J Clin Transl Sci. 2024 Oct 3;8(1):e137. doi: 10.1017/cts.2024.609 (PMC11523010; doi:10.1017/cts.2024.609)
Supplement: Mora et al. supplementary material [file S2059866124006095sup001.docx]

**Supplemental Material.**

**Supplement Appendix 1.** Dissemination Advisory Board Membership & Expertise

Purpose: to share insights, experiences, and suggestions to assist in the development of a

dissemination strategy to bring the ENACT Network to clinical investigators in the CTSA.

consortium, along with other members of the Dissemination Advisory Board.

| Anne Coughlan, PhD | Polk Bros. Chair in Retailing, Professor of Marketing, Kellogg School of Business, Northwestern University.  A nationally recognized expert on the strategic development and use of complex distribution channels to bring products to end users (and author of A Field Guide to Channel Strategy). |
| --- | --- |
| Jim Dearing, PhD | Professor and Chair, Department of Communications, Michigan State University.  Nationally- recognized NIH-funded expert on diffusion of innovations, including adoption and implementation of new evidence-based practices, programs, and  technologies in a healthcare setting. |
| Deborah Goeken, MPH | Vice-President, Colorado Health Institute (retired).  Expert in strategic communications and messaging in health and health care; former managing director of the Rocky Mountain News, a Pulitzer Prize-winning publication. |
| Jerry Shelton, R.Ph | Retired executive, Merck & Co, Inc.  Expert in domestic and international product launch and marketing management in the pharmaceutical industry. |
| Wayne Guerra, MD, MBA | Co-founder/CMO, iTriage, CU Entrepreneur-in-Resident  Serial healthcare entrepreneur experienced in consumer-facing mobile and digital health technology, including business model creation, product development, and customer acquisition/retention. |
| Charles P. Friedman, PhD | Department Chair of Learning Health Sciences Josiah Macy Jr. Professor of Medical Education, Professor of Information, Professor of Public Health at the University of Michigan.  Expertise in Informatics and Learning Health Systems. |

**Supplement Appendix 2.** Customer Discovery Interview and Focus Group Guide

Customer Discovery Interview Guide

I. I am…

I am not here to sell you anything, I’m here to learn from you.

• My name is and a short background on YOU, not your tech.

• If you are interested, we can give you an idea at the end.

• We are part of a program sponsored by the NCATS CTSA focused on understanding your needs to improve ACT. It has been great!

II. May I…

• May I have about 25 minutes of your time, does that still work for you?

• I’m going to ask only a few questions. May I take notes? Record? Take a picture of you? Use your name for the class?

• I am here with my teammate, are you OK if they listen in?

• I’d like to use the information you provide for this purpose, OK?

• I’ll blind all _____ information if needed. We cannot sign an NDA however and we stick to non-confidential information.

III. You are…

• You are a friend of? A graduate of university? Work with?

• I have you as title, affiliation, etc.

• I read the paper/blog/article you published and thought …

• The person who referred me to you said you were an expert in this field _______.

IV. Tell me…

• A little about your day and how often you might do this task.

• I think that task is provided to you by _______. Can you tell me how it occurs in your setting?

• Who else is involved in the decision-making processes?

• When did you start doing it that way? Why?

V. Barriers and Facilitators.

• What’s working well for you?

• What makes you happy here?

• What’s not working well?

• Why?

• Tell me more…

• If you were king or queen for a day what would you like to see happen?

VI. Final thoughts.

• Is there anything I should have asked about and didn’t?

• If I have more questions can I follow up later?

• Who else should I speak with?

• Can you help me connect?

Focus Group Guide.

This was a structured set of four questions listed below. To the extent possible, individuals were queried, and then open discussion ensued around selected topics.

1. What excites them individually for what is coming in health informatics over the next five years?

2. What are the barriers to achieving those goals?

3. How does ACT/ENACT fit into that future?

4. How does the translation between EHR research and the clinic get made?

**Supplement Appendix 3.** Competitive Landscape Analysis EHR Data Networks

This table presents an analysis of electronic health data networks available for research use in the United States as of 2023. They have been stratified by the type of data and cost per data acquisition. Cost data were derived from the website information available. Networks have been color-coded based on their data governance structure. Open indicates access to the public without restrictions; Membership, accessible for those who belong to the specific data network; DUA/LA, Data use agreement/License agreement; and Hybrid, depending on the dataset requested requirements for accessibility vary.


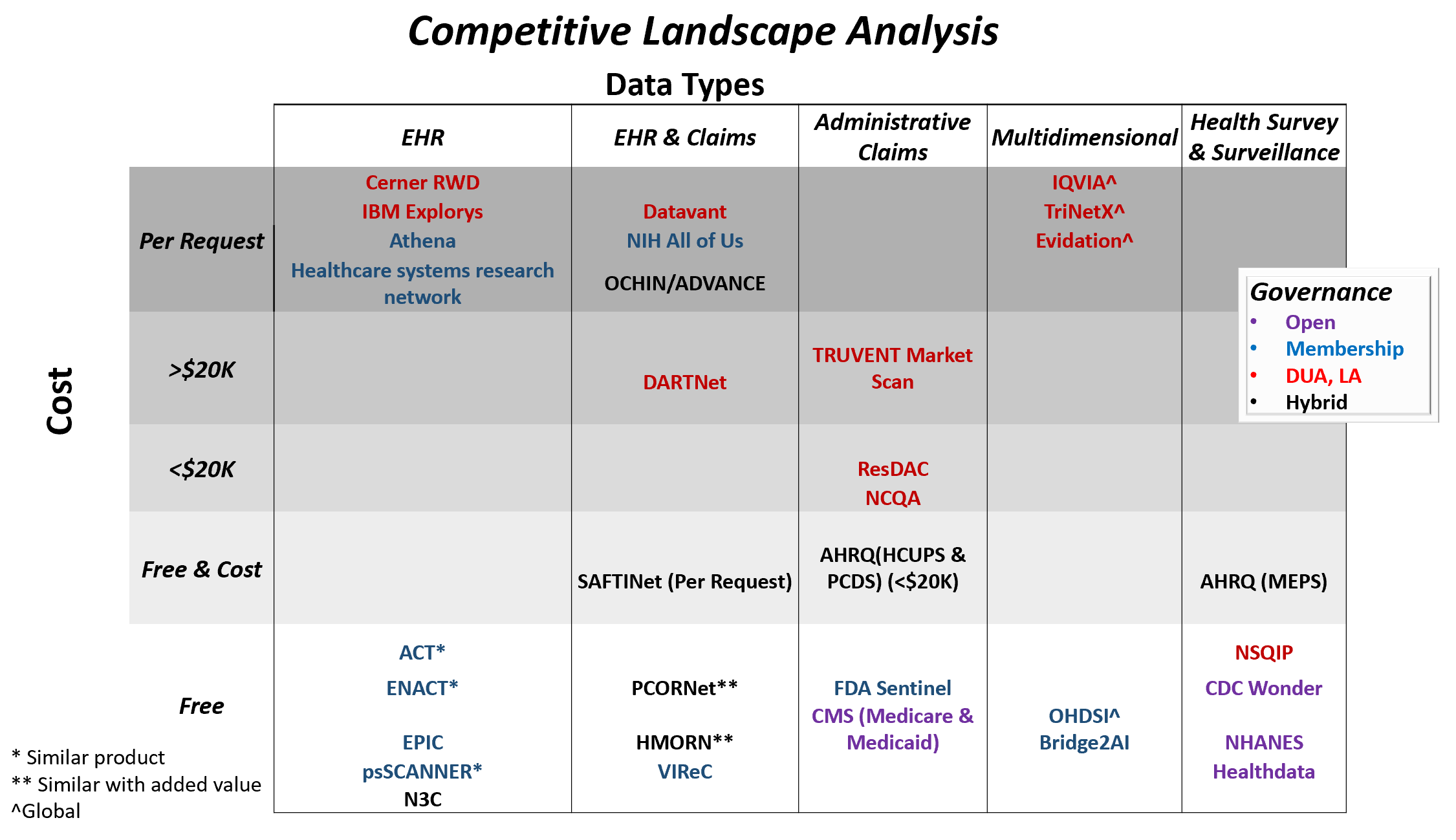


**Footnote:**

IPSOS-[Ipsos Group S.A.](https://www.ipsos.com/en/healthcare-evidence-value-and-access), IRI’s MULO- Multioutlet (now [CIRCANA](https://www.circana.com/)) , [NielsenIQ-](https://nielseniq.com/) , EPIC- [Epic Systems Corporation](https://www.epic.com/), pSCANNER- Patient center scalable national network for effectiveness research, [IBM Explorys](https://www.ibm.com/case-studies/smartanalyst-oncology/?mhsrc=ibmsearch_a&mhq=Explorys), Cerner Data Centre- now [Oracle](https://www.oracle.com/health/cloud/), Athena- [Athenahealth](https://loyolauniversitychicago-my.sharepoint.com/personal/namora_luc_edu/Documents/ENACT/Customer%20Discovery/Publication%20Draft/Last/athenahealth.com) EHR and medical billing solution, HSRN- [High Speed Research Network](https://www.nyu.edu/life/information-technology/research-computing-services/research-data-and-tools/high-speed-research-network-hsrn.html) , ResDAC- [Research Data Assistance Center](https://resdac.org/), NCQA- [National Committee for Quality Assurance,](https://loyolauniversitychicago-my.sharepoint.com/personal/namora_luc_edu/Documents/ENACT/Customer%20Discovery/Publication%20Draft/Last/ncqa.org) Truven Market Scan- now [IBM MarketScan Databases](https://pophealth.ucsf.edu/ibm-marketscan-research-databases-access), HMORN- Health Medical Research Network virtual Data Warehouse, PHIS- [Pediatric Health Information System](https://www.childrenshospitals.org/content/analytics/product-program/pediatric-health-information-system), [DARTNet](https://dartnet.info/ResearchAndData.htm), OCHIN- [OCHIN-led ADVANCE research network,](https://ochin.org/) [Datavant](https://www.datavant.com/), ODSHI-[The Observational Health Data Sciences and Informatics](https://www.ohdsi.org/), [Bridge2AI](https://www.commonfund.nih.gov/bridge2ai), [TriNetX](https://trinetx.com/), [Evidation](https://evidation.com/), [IQVIA](https://www.iqvia.com/), NHANES-National Health and Nutrition Examination Survey, AHRQ(MEPS)- Agency for Healthcare Research and Quality-[Medical Expenditure Panel Survey](https://www.ahrq.gov/data/meps.html), CDC Wonder- Center for Disease Control Wide-ranging ONline Data for Epidemiologic Research, FDA Sentinel- Federal Drug Administration Sentinel Initiative, [CMS-Centers for Medicare & Medicaid Services](https://www.cms.gov/data-research), AHRQ(HCUP)- Agency for Healthcare Research and Quality-[Healthcare Cost and Utilization Project](https://www.ahrq.gov/data/hcup/index.html), AHRQ (APCDs)-Agency for Healthcare Research and Quality-[All-payer claims databases](https://www.ahrq.gov/data/apcd/index.html) , ACT-Accrual to Clinical Trials, N3C-National COVID Cohort Collaborative, ENACT- Evolve to Next-Gen ACT Network, PCORnet-National Patient-Centered Clinical Research Network, VIReC- Veterans Affairs Information Resource Center, SAFTINet-Scalable Architecture for Federated Translational Inquiries Network, NIH All of Us- National Institute of Health All of Us Research Program.

**Supplement Appendix 4.** Table of U.S. National EHR Data Network Products and their Attributes

| **Name** | **Data source** | **Data Type** | **Scale** | **Governance** | **Regulation** | **Cost** | **Open Access (Y/N) *** | **Data model (OMOP, Leaf)** |
| --- | --- | --- | --- | --- | --- | --- | --- | --- |
| Accrual to Clinical Trials (ACT) | EHR | Care setting | National | Available to participating CTSAs | Local DUA | Free | No | i2b2 |
| pSCANNER (patient-centered SCAlable National Network for Effectiveness Research) | EHR | Clinical data |  | Accessible to affiliated sites | DUA, IRB | Free | No | OMOP common data model |
| PCORnet | EHR | EHR linked to payor data | National | Access to partners and per request by authorization | DUA | Free, but needs proof of sustainability | No | PCORnet data model |
| HMORN (HMO research network) | EHR | EHR linked with medical claims | National (19 sites) | Access to partners and per request by authorization | DUA | Free & cost | No | Distributed data model |
| SAFTINet | EHR | EHR linked w/ medical claims | National | Access to partners and upon request & fee to others |  | Free & cost (custom base $10,000, standard $13K) | Public & private | OMOP |
| ENACT | EHR | Care setting | National | Access to CTSIs affiliated partners | DUA | Free | No | i2b2 |

*Open Access refers to governance structure, the alternatives is by membership or data use agreements (DUA) or licence agreements (LA).

SAFTINet- SAFTINet-Scalable Architecture for Federated Translational Inquiries Network, ENACT- Evolve to Next-Gen ACT Network, EHR- Electronic Health Records, DUA- Dta User Agreement, IRB- Institutional Review Board, i2b2- Informatics for Integrating Biology & the Bedside, OMOP- Observational Medical Outcomes Partnership (OMOP) Common Data Model (CDM), PCORnet®- the National Patient-Centered Clinical Research Network.

**Supplement Appendix 5.** Ecosystem map- Research use of electronic health data at academic CTSA institutions by workflow stage**.**

**Workflow and Decision Making within medium to large institutions**

| **Role** | **Idea & Feasibility** | **Data Sourcing** | **Interaction with the Core facility group** | **Rescoping of Data Sources** | **Acquisition, Analysis, and Publication** |
| --- | --- | --- | --- | --- | --- |
| Activity by researcher | Rough out of project and cohort size consideration | Considers local, regional, or other familiar databases | Interaction begins and detailed project discussion starts | Now understands the benefits of rescoping | Data is acquired by Core and analysis begins. Duration depends. |
| Influencers | Collaborators and research lines | Collaborators, possibly Core facilities | Core and informatics leads, stats | Project leads from Core Facility | Statistics |
| Problems encountered |  |  | To use or not to use… | Discomfort from being not in control |  |

*Influencers- The people who influence the local business model and data governance necessary to advance local health informatics research
